# Asthma/Rhinitis (The United Airway) and Allergy: Chicken or Egg; Which Comes First?

**DOI:** 10.3390/jcm9051483

**Published:** 2020-05-14

**Authors:** John O. Warner

**Affiliations:** 1 Emeritus Professor of Paediatrics, National Heart and Lung Institute, Imperial College, London SW3 6LY, UK; j.o.warner@imperial.ac.uk; 2 Honorary Professor of Paediatrics, University of Cape Town, Cape Town 7701, Western Cape, South Africa

**Keywords:** asthma, rhinitis, allergy, allergic sensitization, genomics, epigenetics, hygiene hypothesis, allergic march, gene/environment interactions, personalized medicine

## Abstract

While allergy, asthma and rhinitis do not inevitably co-exist, there are strong associations. Not all those with asthma are allergic, rhinitis may exist without asthma, and allergy commonly exists in the absence of asthma and/or rhinitis. This is likely due to the separate gene/environment interactions which influence susceptibility to allergic sensitization and allergic airway diseases. Allergic sensitization, particularly to foods, and eczema commonly manifest early in infancy, and not infrequently are followed by the development of allergic rhinitis and ultimately asthma. This has become known as the “allergic march”. However, many infants with eczema never develop asthma or rhinitis, and both the latter conditions can evolve without prior eczema or food allergy. Understanding the mechanisms underlying the ontogeny of allergic sensitization and allergic disease will facilitate rational approaches to the prevention and management of asthma and allergic rhinitis. Furthermore, a range of new, so-called biological, therapeutic approaches, targeting specific allergy-promoting and pro-inflammatory molecules, are now in clinical trials or have been recently approved for use by regulatory authorities and could have a major impact on disease prevention and control in the future. Understanding basic mechanisms will be essential to the employment of such medications. This review will explain the concept of the united airway (rhinitis/asthma) and associations with allergy. It will incorporate understanding of the role of genes and environment in relation to the distinct but interacting origins of allergy and rhinitis/asthma. Understanding the patho-physiological differences and varying therapeutic requirements in patients with asthma, with or without rhinitis, and with or without associated allergy, will aid the planning of a personalized evidence-based management strategy.

## 1. Introduction

There is a compelling list of evidence to support the concept that allergy is fundamental to persistent asthma. Allergy in the child and family is a risk factor for later rhinitis and asthma. Early onset allergy is a poor prognostic factor for those who subsequently develop asthma. Direct allergen exposure into the nose in allergically sensitized subjects will incite allergic rhinitis and inhaled into lower airways an asthmatic reaction. The severity of asthma is directly associated with the degree of allergy and with food allergy. Monoclonal anti-Immunoglobulin E (IgE) improves allergic asthma and allergen immunotherapy is the only treatment which modifies long-term outcomes for rhinitis and asthma. However, the attributable percentage of asthma due to allergy never reaches even 50%, and in some environments is less than 10%. The overall attributable fraction throughout Europe from one study, was 30%, but ranged between 4% and 61% between countries [1]. However, attribution varies with age, with allergy being more strongly related to asthma in children compared to adults.

The histopathology of asthma, and to a lesser extent allergic rhinitis, is characterized by airway inflammation, involving to a variable extent, neutrophils, eosinophils and mast-cells. However, the component most associated with persistent and more severe disease is airway remodeling, with increased collagen in the lamina reticularis (seen as apparent thickening of the basement membrane) and submucosa. There is also hypertrophy of smooth muscle and associated bronchial hyper-responsiveness (BHR). Many of these features sometimes manifest independent of allergy [2].

It is therefore necessary to re-evaluate the allergy/rhinitis/asthma relationships.

## 2. Gene/Environment Interactions

The genetics of rhinitis and asthma do not exhibit simple Mendelian inheritance. Many genes are involved, each having very small phenotypic effects. Gene polymorphisms have variously been associated with allergy, and/or asthma, and/or eczema, and/or rhinitis. It has therefore been suggested that it is more fruitful to focus on gene/environment interactions, epigenetics, and pharmaco-genetics [3].

**Figure 1:**
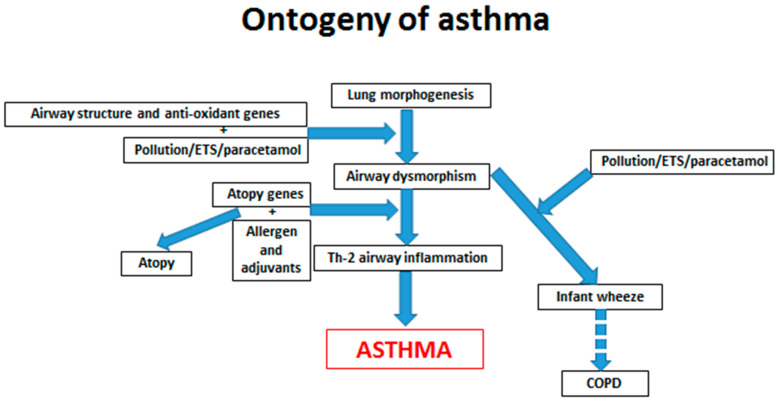
A schematic representation of the impact of timing of environmental interactions with genetic polymorphisms on the ontogeny of allergic sensitization and three wheeze phenotypes; infant wheeze, asthma and Chronic Obstructive Pulmonary Disease (COPD). ETS is environmental tobacco smoke.

Airway inflammation and bronchospasm can occur because of gene and environmental interactions, resulting in alterations in airway structure and function (airway dysmorphisms), independent of allergy (Figure 1). Changes in structure can modify the behavior of inflammatory cells and susceptibility to infection. Thus, polymorphisms in the A Disintegrin and Metalloprotease 33-ADAM 33 (20p13) and OrmDL3 (17q21) genes increase susceptibility to wheezing in infancy and possibly later-life Chronic Obstructive Pulmonary Disease—COPD, but not necessarily asthma. These genes are not expressed in immunologically active cells but are associated with influences on airway structure and function [4,5]. There is evidence that ADAM 33 polymorphisms result in airway remodeling in the presence of environmental triggers, and that this may even commence during fetal life [6]. A meta-analysis of genome-wide association studies has shown linkage with several genes on chromosome 17q21, including those coding for Thymic Stromal Lymphopoetin (TSLP) and Interleukin-33 (IL33), which are expressed in epithelial cells. When these cells are damaged, TSLP, IL25 and IL33 are released and stimulate release of IL4 from innate lymphocytic cells. This in turn facilitates the development of an adaptive T-helper lymphocyte type 2 (Th2) allergy promoting hypersensitivity [7]. Implicit from all the association studies is that asthma is heterogeneous with a range of genetic influences on airway morphology, and on signals from damaged epithelial cells which switch on an adaptive immune response leading to airway inflammation. These changes are not influenced by prior allergic sensitization but may contribute to its subsequent development [8]. 

Gene polymorphisms, which impair anti-oxidant activity, predispose to wheeze in the presence of early life environmental tobacco smoke (ETS) and other pollutant exposures, as well as airway infections, but are not necessarily associated with allergy [9]. Epidemiological studies have suggested that both maternal pregnancy and infant paracetamol exposure increase the risk of asthma [10,11]. A paracetamol metabolite, n-acetylbenzoquinonimine, depletes anti-oxidant activity, and one intriguing study suggests that there is an interaction, increasing the risk of wheeze at 5 years of age, between the number of days that pregnant women take this analgesic and a polymorphism in the Glutathione S-Transferase P-1 anti-oxidant gene [12]. Impairment of anti-oxidant activity will increase the risks of persistent airway inflammation in response to infection or pollutant exposure.

Separate gene polymorphisms affecting immune modulation predispose to allergic sensitization in the presence of allergen and adjuvant exposure. There are fewer genome-wide association studies of allergic sensitization alone compared with those for allergic diseases such as asthma and eczema. They have revealed a range of gene polymorphisms, associated with regulation of T-lymphocyte responses, IgE (the allergy antibody) production, and the IgE receptor [13]. Some are common to those associated with asthma, such as genes for TSLP and IL33, while others are independent, such as those in the cytokine gene cluster on 5q31-33 [14].

Variations in the genome (nature) have only small effects on allergy and airway phenotypes, and environment (nurture) clearly plays a greater part. Epigenetic phenomena explain much of the science underlying gene/environment interactions. These are changes to DNA expression without modification of DNA sequences. They occur by: methylation of DNA Cytosine-phosphodiesterase-Guanine CpG motifs (30 million in the human genome), which impairs transcription; histone modification, around which DNA is coiled, on 30 million nucleosomes by acetylation, methylation, phosphorylation, and ubiquitination, which opens chromatin to aid transcription; and microRNAs in the cytoplasm, which mostly block mRNA transcription. Many environmental factors have their influence through epigenetic effects. This has been best studied for CpG methylation [15]. Most notable is the effect of ETS and pollutant exposure, which have been shown to affect CpG methylation and histone modification [16]. While influences may occur at any age, epigenetic effects can be hereditable, and this has been demonstrated in a study of grand–mother ETS exposure which independently increased the risk of asthma in grand-children, irrespective of maternal smoking [17]. Even minor variations in diet can have epigenetic effects. Thus, folic acid—a methyl donor—supplementation during pregnancy has been associated with an increased risk of asthma in childhood [18].

Pharmaco-genetic studies have identified genetic variations which result in different responses to medications. Beta-2 adrenergic receptor polymorphisms have been associated with beta-receptor sub-sensitization if beta-2 agonists are administered continuously in the absence of cortico-steroids, leading to increased BHR and deteriorating asthma control. Polymorphisms in the 5-lipoxigenase genes result in lack of improvement when treated with the leukotriene receptor antagonist, Montelukast. With the advent of personalized medicine, these pharmaco-genetic insights will aid decisions on therapeutic approaches to asthma management [19].

## 3. Wheeze Phenotypes

**Figure 2:**
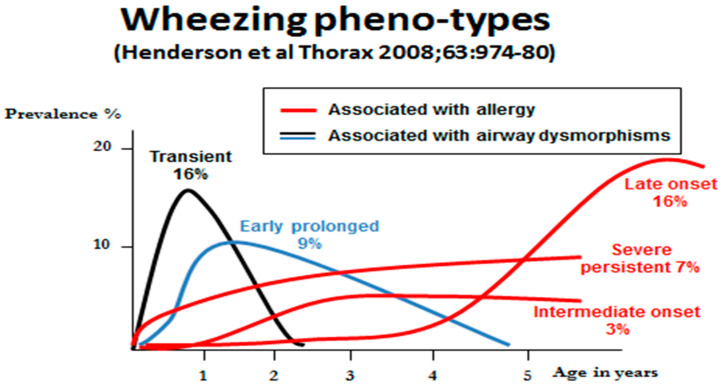
A graphic representation of data from the Avon longitudinal birth cohort study [20], showing the distributions of five wheeze phenotypes over the first five years of life.

Based on longitudinal birth cohort studies, several distinct wheezing phenotypes have been characterized, each of which have different clinical courses [20]. The understanding emerging from studies of gene environment interactions, as outlined above, begin to explain the underlying mechanisms involved with each phenotype (Figure 2). Early life transient and more prolonged pre-school wheezing is related to gene/environment interactions adversely affecting airway form, function and susceptibility to infection. Indeed, infant wheeze is often preceded by airway function deficits detectable at birth and commonly related to maternal pregnancy smoking [21]. The same factors may be associated with adult onset non-allergic asthma and COPD [6]. More persistent wheeze in childhood beyond 3–5 years of age is mostly associated with allergy.

## 4. Early Life Origins of Allergy

Studies of the ontogeny of allergy have increasingly focused on the first 1000 days, from conception to 2 years of age. There is a sequence of events which interact to affect the relative risk of allergy development. The decidual tissues around the fetus promote a Th2 and T-cell regulatory environment through the generation of IL4, IL13, and Transforming Growth Factor beta (TGFβ), which are normal components of the mechanisms generating protection against a maternal Th1 response to fetal–paternal antigens [22]. Absence of this regulation is associated with recurrent early miscarriage and intra-uterine growth retardation, in both murine models and humans [23]. The fetus has the capacity to switch on adaptive immune responses via lymphoid accumulations in the small bowel from the middle of the second trimester of pregnancy [24]. Fetal allergen exposure, in association with Th2-promoting cytokines, occurs via swallowed amniotic fluid in the second trimester, and antigen presentation and sensitization can occur through the fetal gut. Consequently, all neonates have a Th2-biased immune response, which is more entrenched when the mother is also allergic [25]. Balancing of the response occurs in the third trimester, through allergen aggregated with IgG transported across the placenta, which down-regulates sensitization through inhibitory IgG receptors. This has been demonstrated by studies showing strong associations between raised IgG antibodies to both ingestant and inhalant allergens in cord blood, and a lower subsequent risk of allergic sensitization [26,27]. One remarkable observational study showed that children of mothers who had undergone rye-grass allergen immunotherapy during pregnancy, and consequently had high IgG antibody levels, compared to children born to rye-grass allergic mothers not receiving immunotherapy, showed fewer positive allergy skin tests to rye-grass 3–12 years later in the offspring [28].

Likely the most important post-natal influence balancing the immune response is diversification of the neonatal microbiome. This explains the association between Caesarean section delivery, or maternal pregnancy antibiotic exposure, and increased risks of food allergy in the offspring [29,30]. In both circumstances, the infant is not exposed to a normal maternal microbiome, by either avoiding passage through the birth-canal or encountering one modified by the antibiotics. Additional and potentially critical reinforcement of neonatal microbiome diversification is provided by a range of factors, including pre-biotic oligosaccharides in human breast milk [31]. Rapid diversification of the gut microbiome is associated with a lower risk of later allergic disease [32]. The post-natal gut and liver in the presence of a normal microbiome, in the absence of co-stimulatory signals, induce tolerance to swallowed antigens/allergens. Later in infancy, early admission to day care, or having an older sibling, is associated with increased early minor respiratory infections, but reduced later risk of allergic disease. This was the origin of what became known as the “hygiene hypothesis”, now better described as the “microbial exposure hypothesis” [33].

## 5. Prevention and Treatment of Allergy

Based on an understanding of the ontogeny of allergy, it is likely that allergen avoidance will have no role in primary prevention of allergic sensitization. Indeed, several studies both in relation to food and inhalant allergy have indicated that early high exposure, from conception through the first months of life, may reduce the later risk of allergic sensitization and disease. Increased levels of IgG-specific antibodies in cord blood, reflecting high maternal exposure to food and inhalant allergens, are transferred across the placenta from mother to fetus in the last trimester of pregnancy, and are associated with a lower risk of later allergic sensitization in infants [26,27,28]. There is not a linear relationship between exposure and allergy. There is a bell-shaped curve, with low levels being insufficient to switch on a response, while high levels induce immune tolerance by creating anergy or deletion of sensitized T-lymphocytes. Intermediate exposure is more likely to be associated with sensitization and the evolution of allergic disease [34].

Aero-allergen exposure, in those who have rhinitis and/or asthma and are already allergically sensitized, induces immediate (within minutes) mast-cell degranulation, and thereby sneezing, rhinorrhoea, coughing and wheezing. In those with more severe disease, a late inflammatory reaction, dominated by first neutrophils and then eosinophils, evolves three to four hours later. The immediate response is self-limiting, and can be completely reversed by antihistamines for rhinitis and bronchodilators for asthma, while the late reaction is poorly anti-histamine/bronchodilator responsive and is only abrogated by prior treatment with inhaled (or oral/parenteral) cortico-steroids (ICS). The late reaction occurs more frequently in those with more severe airway disease [34]. This provides a basis for understanding the place of each medication in a therapeutic strategy.

Implicit to the knowledge that allergen exposure aggravates pre-existing allergic airway disease is the concept that allergen avoidance will be of benefit. However, many trials have failed to demonstrate consistent efficacy, particularly in relation to house dust mite (HDM) allergic asthma/rhinitis. This has led to Cochrane systematic reviews, reaching the conclusion that allergen avoidance has no place in therapeutic algorithms [35]. The difficulty is that exposures occur in many places, and strategies to reduce aero-allergen levels, whether to HDM, animals, pollens or molds, have been insufficient. There is evidence that perfect avoidance, such as in high altitude environments, as in the Alps, can have considerable benefits [36]. Furthermore, recent trials of an effective environmental control system, based on temperature controlled laminar airflow, employed overnight, have shown significant improvements in quality of life and eosinophilic airway inflammation (represented by raised exhaled nitric oxide levels) in severe asthma in children and adults [37,38]. While guidelines for asthma management have tended to consider allergen avoidance as secondary to pharmaco-therapy [39], those for rhinitis have given this approach a higher priority [40].

## 6. The Allergic March

**Figure 3:**
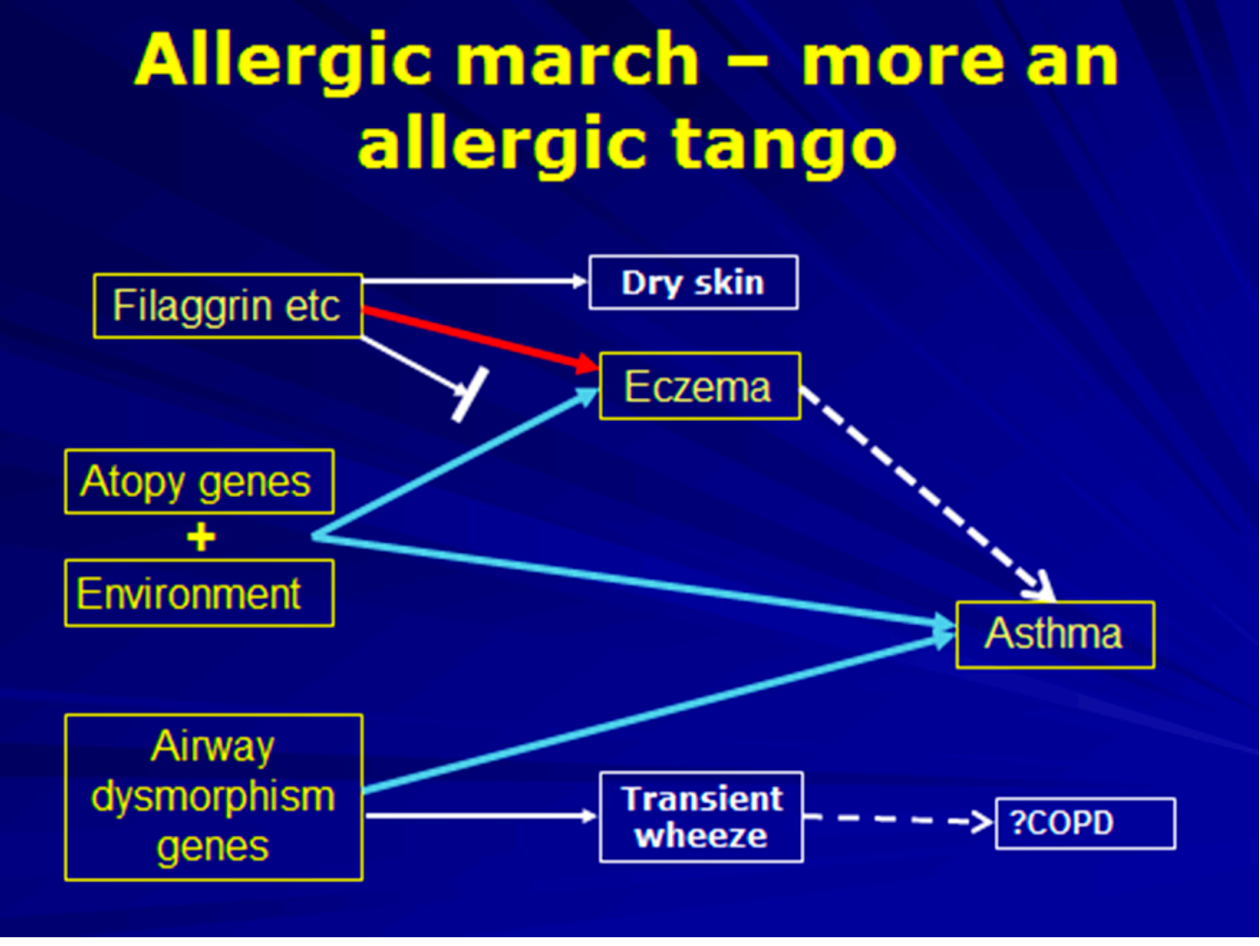
An illustration of the complex relationships between various allergic phenotypes. Constituting not an allergic march but more a complex dance such as the tango.

The usual representation of the allergic march presents food allergy, followed in succession by eczema, asthma, and finally hay fever. The implication is that one condition leads inexorably to another. However, as our understanding of the genetic basis of eczema and asthma has increased, it has become clear that there are independent influences on each. Nevertheless, there are mechanistic explanations for progression through the march for a proportion of subjects. The genetic basis for eczema is a defect in skin barrier function, of which the best known is polymorphisms in the gene coding for filaggrin. Defects in this protein lead to increased fluid losses from the epidermis, and greater susceptibility to inflammation induced by irritants and micro-organisms, thereby leading to eczema. The defect also renders the skin more susceptible to allergen penetration into the dermis, where antigen-presenting cells can pick up allergens and migrate to regional lymphoid accumulations for sensitization to occur [41]. Thus, the sequence of the march is incorrect, with food allergy a consequence of the gene defect contributing to eczema, and more likely to follow rather than precede it. Inhalant allergy may also evolve by the same route and increase the risk of rhinitis and asthma, though likely only if there is also airway dysmorphism. This could be viewed as a true march, though it may be better to view the overall allergic diseases’ associations as a complex stop/start dance—Figure 3 [42].

The other discrepancy in the conventional representation of the march is that rhinitis is more likely to precede rather than follow the development of asthma. This is very clear in the evolution of occupational allergic airway disease. Sensitization to the occupational allergen is followed by rhinitis and skin manifestations. Asthma develops after more sustained exposures. Provided avoidance is instituted early, asthma can be prevented or completely resolved. However, if exposure continues, eventually asthma becomes entrenched and will continue even if the occupational allergen is avoided [43]. If this sequence occurs in allergic asthma, as is very likely, then early avoidance of the primary allergic trigger could have much greater benefit than has been achieved once asthma has reached a chronic stage, with structural (remodeling) changes to the airway wall. This approach requires more research trials.

## 7. Pharmaco-Therapy for United Airway Diseases

The nose should be considered as the most accessible segment of the respiratory tract. The epithelial lining is identical throughout the conducting airways, and the histo-pathology of allergic inflammation is very similar. From a German longitudinal study, amongst those with allergic rhinitis before 5 years of age, the relative risk for asthma from 5–13 years of age is increased at 3.82%, and 41.5% of children aged 5–13 with new onset wheezing had preceding allergic rhinitis [44]. Conversely, allergic rhinitis co-exists in at least 85% of those with asthma, but is commonly not detected. Perhaps more importantly, treatment of the rhinitis significantly improves asthma control [45].

Mild intermittent airway disease can be treated with non-sedating antihistamines for rhinitis, and bronchodilators as necessary for wheeze. Persistent disease must be treated with regular ICS to achieve control. For those with poor asthma control, long-acting beta agonists (LABAs) and/or leukotriene receptor antagonists (LtRAs) can be added to ICS [39,40]. For the most severe disease, new pharmacological and biological agents are beginning to gain regulatory approval (see following section).

## 8. Allergen Immunotherapy

While Cochrane systematic reviews have established that subcutaneous allergen immunotherapy improves both rhinitis and asthma, the potential for life-threatening anaphylactic reactions in those with severe asthma means that, at present, its only recommended use in the UK is for allergic rhinitis and insect venom allergy [46]. Its use in allergic rhinitis is clearly established [40]. Furthermore, there is increasing evidence that this approach to management is the only one which truly modifies the natural history of allergic airway disease. Pollen immunotherapy, administered over three consecutive years, had a prolonged carry-over effect for several years after stopping treatment [47]. Furthermore, allergen immunotherapy for three years in children with allergic rhinitis alone reduced the subsequent risk of developing new allergies and asthma [48,49]. The advent of sub-lingual and epidermal vaccines, and modification of allergens, including immunogenic allergen peptides, is beginning to change practice [50]. These beneficial effects of treatment directly addressing allergy demonstrate that allergy has a major impact on asthma severity as an inciter, even if it is not the cause (inducer) of disease. With increasing evidence of safety and efficacy, it is likely that allergen immunotherapy will be used much more extensively in the future.

## 9. New Approaches to Management of Allergic Airway Diseases

The very worst allergic airway disease can be improved with monoclonal Anti-IgE, administered by sub-cutaneous injections (Omalizumab) [51]. This is the first biological agent to be approved for use in severe asthma. It binds circulating IgE, thereby preventing its attachment to IgE receptors, which consequently down-regulate with long-term treatment. So far there is no evidence that this therapy modifies long-term outcomes in the same way as allergen immunotherapy. It has also been employed, with benefit, in allergic rhinitis, severe eczema, persistent urticaria, and as an adjunct to allergen immunotherapy, however its high costs preclude use other than in the most severe forms of allergic disease [52].

More recently, monoclonal antibodies have been developed which target Interleukin 5 (IL5) or its receptor, which is a key cytokine in mediating eosinophilic inflammation. Amongst these, Mepolizumab, Reslizumab and Benralizumab have been approved by the National Institute for Health and Clinical Excellence for use in severe eosinophilic asthma [53].

Mono-clonal antibodies have been developed which block the activity of IL 4 and 13, which are key cytokines inducing much of the pathology of allergic disease. One such product, Dupilumab, blocks the alpha sub-unit of the IL4 receptor, thereby inhibiting the effects of both IL4 and IL13. It has been shown to improve severe asthma and eczema [54].

Prostaglandin D2 receptors on Th2 lymphocytes mediate much of the allergic inflammatory response. Fevipiprant is an orally active inhibitor of this receptor, which has had variable beneficial effects for eczema and asthma [54].

These new developments make understanding of basic mechanisms an imperative to utilizing novel biological agents, personalized to what has become known as the patient’s endo-type (combination of clinical and immunological characteristics).

## 10. A Shared Misunderstanding between Patient and Physician

The most important component of management is the development of an accord between physician, patient and carer, to achieve concordance with the treatment plan. Patients and carers have first-hand experience of the triggers of exacerbations of airway symptoms. They expect that clinicians will aid identification and confirmation that such triggers are relevant. While it is incumbent on clinicians to address the concerns of their patients, many do not believe that testing for allergic triggers is either necessary or likely to make a difference to treatment. This is often based on the negative Cochrane review of HDM avoidance. The failure to even consider the patient’s perception of their disease is likely to lead to a mutual misunderstanding, where an accord has not been reached and concordance with therapeutic recommendations will not be achieved [55]. Furthermore, allergy, to foods and inhalant allergens, is a common association with life-threatening and fatal asthma. Lack of attention to allergic triggers will therefore not only affect concordance but could have severe adverse consequences. My view is that all patients with rhinitis and asthma should have a full allergy-focused history and should be tested for relevant allergy triggers.

Patient/carer education and training, particularly in the use of inhaler devices and monitoring of disease control, is imperative. There must be clear guidance on how to recognize loss of disease control and what action to take. A written, personalized, mutually agreed asthma action plan must be formulated to aid on-going treatment and provide a strategy in event of deterioration in control. Any exacerbation must be considered a failure of control, with the need for immediate review to establish the cause and adjust management accordingly [56].

## 11. Conclusions

Asthma and rhinitis are commonly associated with allergy, particularly in younger age groups. While allergy and subsequent allergen exposure exacerbates pre-existing airway disease, it is unclear to what extent allergic sensitization is a disease-inducer. Recent insights into the gene-environment interactions suggest that there are often independent influences on allergy and airway disease susceptibility. In some circumstances, changes in airway morphology precede the development and may even facilitate allergic sensitization. In other cases, allergy causes changes in airway morphology, leading to rhinitis and asthma, most obviously with occupational allergen exposures. Therefore, the analogy of which comes first—the chicken or egg—is a circuitous argument. Nevertheless, understanding the early life origins and basic mechanisms will aid the employment of the most appropriate management strategies. Evidence suggests that allergen avoidance to prevent allergic sensitization, which constitutes primary prevention, is ineffective and based on immunological insights is an inappropriate strategy. However, addressing allergy either by avoidance or allergen immunotherapy is an important component of management of those already allergically sensitized, and can be viewed as secondary prevention. Clinicians ignoring any one component of the triad (rhinitis, asthma and allergy) do so at their own, and their patients’, peril.

## References

[1] Sunyer J., Jarvis D., Pekkanen J., Chinn S., Janson C., Leynaert B., Luczynska C., Garcia-Esteban R., Burney P., Antó J.M. (2004). Geographic variations in the effect of atopy on asthma in the European Community Respiratory Health Study. J. Allergy Clin. Immunol..

[2] Warner J.O., Boner A.L., Holgate S.T., Church M.K., Broide D.H., Martinez F.D. (2012). Paediatric Allergy and Asthma. Allergy.

[3] Collins S.A., Lockett G.A., Holloway J.W., Leung Y.M., Sampson H.A., Geha R.S., Szefler S.J. (2016). The genetics of allergic diseases and asthma. Pediatric Allergy Principles and Practice.

[4] Van Eerdewegh P., Little R.D., Dupuis J., Del Mastro R.G., Falls K., Simon J., Torrey D., Pandit S., McKenny J., Braunschweiger K. (2002). Association of the ADAM33 gene with asthma and bronchial hyper-responsiveness. Nature.

[5] Moffatt M.F., Kabesch M., Liang L., Dixon A.L., Strachan D., Heath S., Depner M., von Berg A., Bufe A., Rietschel E. (2007). Genetic variants regulating ORMDL3 expression contribute to the risk of childhood asthma. Nature.

[6] Davies E.R., Kelly J.F.C., Howarth P.H., Wilson D.I., Holgate S.T., Davies D.E., Whitsett J.A., Haitchicorresponding H.M. (2016). Soluble ADAM33 initiates airway remodeling to promote susceptibility for allergic asthma in early life. JCI Insight..

[7] Torgerson D.G., Ampleford E.J., Chiu G.Y., Gauderman W.J., Gignoux C.R., Graves P.E., Himes B.E., Levin A.M., Mathias R.A., Hancock D.B. (2011). Meta-analysis of Genome-wide Association Studies of Asthma In Ethnically Diverse North American Populations. Nat. Genet..

[8] Moffatt M.F., Gut I.G., Demenais F., Strachan D.P., Bouzigon E., Heath S., von Mutius E., Farrall M., Lathrop M., Cookson W.O.C.M. (2010). A Large-Scale, Consortium-Based Genome wide Association Study of Asthma. N. Engl. J. Med..

[9] Lee Y.-L., Lin Y.C., Lee Y.C., Wang J.Y., Hsiue T.R., Guo Y.L. (2004). Glutathione S-transferase P1 gene polymorphism and air pollution as interactive risk factors for childhood asthma. Clin. Exp. Allergy.

[10] Eyers S., Weatherall M., Jefferies S., Beasley R. (2011). Paracetamol in pregnancy and the risk of wheezing in offspring: A systematic review and meta-analysis. Clin. Exp. Allergy.

[11] Beasley R., Clayton T., Crane J., von Mutius E., Lai C.K., Montefort S., Stewart A., ISAAC Phase Three Study Group (2008). Association between paracetamol use in infancy and childhood, and risk of asthma, rhinoconjunctivitis, and eczema in children aged 6–7 years: Analysis from Phase Three of the ISAAC programme. Lancet.

[12] Perzanowski M.S., Miller R.L., Tang D., Ali D., Garfinkel R.S., Chew G.L., Goldstein I.F., Perera F.P., Barr R.G. (2010). Prenatal acetaminophen exposure and risk of wheeze at age 5 years in an urban low-income cohort. Thorax.

[13] Bønnelykke K., Sparks R., Waage J., Milner J.D. (2015). Genetics of allergy and allergic sensitization: Common variants, rare mutations. Curr. Opin. Immunol..

[14] Yokouchi Y., Nukaga Y., Shibasaki M., Noguchi E., Kimura K., Ito S., Nishihara M., Yamakawa-Kobayashi K., Takeda K., Imoto N. (2000). Significant evidence for linkage of mite-sensitive childhood asthma to chromosome 5q31-q33 near the interleukin 12 B locus by a genome-wide search in Japanese families. Genomics.

[15] Xu C.-J., Söderhäll C., Bustamante M., Baïz N., Gruzieva O., Gehring U., Mason D., Chatzi L., Basterrechea M., Llop S. (2018). DNA methylation in childhood asthma: An epigenome-wide meta-analysis. Lancet Respir. Med..

[16] Munthe-Kaas M.C., Willemse B.W.M., Koppelman G.H. (2012). Genetics and epigenetics of childhood asthma. Eur. Respir. Monogr..

[17] Lodge C.J., Brabak L., Lowe A.J., Dharmage S.C., Olsson D., Forsberg B. (2018). Grandmaternal smoking increases asthma risk in grandchildren: A nationwide Swedish cohort. Clin. Exp. Allergy.

[18] Veeranki S.P., Gebretsadik T., Mitchel E.F., Tylavsky F.A., Hartert T.V., Cooper W.O., Dupont W.D., Dorris S.L., Hartman T.J., Carroll K.N. (2015). Maternal Folic Acid Supplementation during Pregnancy and Early Childhood Asthma. Epidemiology.

[19] Kazani S., Wechsler M.E., Israel E. (2010). The role of pharmacogenetics in improving the management of asthma. J. Allergy Clin. Immunol..

[20] Henderson J., Granell R., Heron J., Sherriff A., Simpson A., Woodcock A., Strachan D.P., Shaheen S.O., Sterne J.A. (2008). Associations of wheezing phenotypes in the first 6 years of life with atopy, lung function and airway responsiveness in mid-childhood. Thorax.

[21] Bisgaard H., Loland L., Holst K.K., Pipper C.B. (2009). Prenatal determinants of neonatal lung function in high-risk newborns. J. Allergy Clin. Immunol..

[22] Warner J.O. (2004). The early life origins of asthma and related allergic disorders. Arch. Dis. Child.

[23] Warner J.O., Gluckman P., Hanson M. (2006). Developmental origins of asthma and related allergic disorders. Developmental Origins of Health and Disease.

[24] Jones C.A., Warner J.A., Warner J.O. (1998). Fetal swallowing of IgE. Lancet.

[25] Jones C.A., Vance G.H.S., Power L.L., Pender S.L.F., MacDonald T.T., Warner J.O. (2001). Costimulatory molecules in the developing human gastrointestinal tract: A pathway for fetal allergen priming. J. Allergy Clin. Immunol..

[26] Jenmalm M.C., Björksten B. (2000). Cord blood levels of immune-globulin-G subclass antibodies to food and inhalant allergens in relation to maternal atopy and the development of atopic disease during the first 8 years of life. Clin. Exp. Allergy.

[27] Vance G.H.S., Grimshaw K.E.C., Briggs R., Lewis S.A., Mullee M.A., Thornton C.A., Warner J.O. (2004). Serum ovalbumin-specific immunoglobulinG responses during pregnancy reflect maternal intake of dietary egg and relate to the development of allergy in early infancy. Clin. Exp. Allergy.

[28] Glovsky M.M., Ghekiere L., Rejzek E. (1991). Effect of maternal immunotherapy on immediate skin test reactivity, specific Rye-1 IgG & IgE antibody and total IgE of the children. Ann. Allergy.

[29] Mitselou N., Hallberg J., Stephansson O., Almqvist C., Melén E., Ludvigsson J.F. (2018). Cesarean delivery, preterm birth, and risk of food allergy: Nationwide Swedish cohort study of more than 1 million children. J. Allergy Clin. Immunol..

[30] Hirsch A.G., Pollak J., Glass T.A., Poulsen M.N., Bailey-Davis L., Mowery J., Schwartz B.S. (2017). Early-life antibiotic use and subsequent diagnosis of food allergy and allergic diseases. Clin. Exp. Allergy.

[31] van den Elsen L.W.J., Garssen J., Burcelin R., Verhasselt V. (2019). Shaping the Gut Microbiota by Breastfeeding: The Gateway to Allergy Prevention?. Front. Pediatr..

[32] Harm Wopereis H., Kathleen Sim K., Alexander Shaw A., Warner J.O., Knol J., Kroll J.S. (2018). Intestinal microbiota in infants at high risk for allergy: Effects of prebiotics and role in eczema developmentin high risk newborns. J. Allergy Clin. Immunol..

[33] Holt P., Naspitz C., Warner J.O. (2004). Early immunological influences in prevention of allergy and allergic disease. Chem. Immunol. Allergy.

[34] Warner J.O., Turner P.J., Lissauer T., Carroll W. (2017). Allergy.

[35] Gøtzsche P.C., Johansen H.K. (2008). House dust mite control measures for asthma. Cochrane Database Syst. Rev..

[36] Peroni D.G., Boner A.L., Vallone G., Antolini I., Warner J.O. (1994). Effective allergen avoidance at high altitude reduces allergen induced bronchial hyperresponsiveness. Am. J. Respir. Crit. Care Med..

[37] Boyle R.J., Pedroletti C., Wickman M., Bjermer L., Valovirta E., Dahl R., von Berg A., Zetterström O., Warner J.O., for the 4A Study Group (2012). Nocturnal temperature controlled laminar airflow for treating atopic asthma: A randomised controlled trial. Thorax.

[38] Warner J.O. (2017). Use of temperature-controlled laminar airflow in the management of atopic asthma: Clinical evidence and experience. Adv. Respir. Dis..

[39] British Thoracic Society/Scottish Intercollegiate Guidelines Network (2016). British Guideline on the Management of Asthma.

[40] Brożek J.L., Bousquet J., Baena-Cagnani C.E., Bonini S., Canonica G.W., Casale T.B., van Wijk R.G., Ohta K., Zuberbier T., Schünemann H.J. (2010). Allergic Rhinitis and its Impact on Asthma (ARIA) guidelines: 2010 revision. J. Allergy Clin. Immunol..

[41] McLean W.H.I. (2016). Filaggrin failure—From ichthyosis vulgaris to atopic eczema and beyond. Brit. J. Derm..

[42] Levin M.E., Warner J.O. (2017). The Atopic Dance. Curr. Allergy Clin. Immunol..

[43] Moscato G. (2017). Occupational allergic airway disease. Curr. Otorhinolaryngol. Rep..

[44] Rochat M.K., Illi S., Ege M.J., Lau S., Keil T., Wahn U., von Mutius E., Multicentre Allergy Study (MAS) Group (2010). Allergic rhinitis as a predictor for wheezing onset in school-aged children. J. Allergy Clin. Immunol..

[45] Welsh P.W., Stricker W.E., Chu C.-P., Naessens J.M., Naessens J.M., Reese M.E., Reed C.E., Marcoux J.P. (1987). Efficacy of Beclomethasone Nasal Solution, Flunisolide, and Cromolyn in Relieving Symptoms of Ragweed Allergy. Mayo Clin. Proc..

[46] Abramson M.J., Puy R.M., Weiner J.M. (2010). Injection allergen immunotherapy for asthma. Cochrane Database Syst. Rev..

[47] Durham S.R., Walker S.M., Varga E.-M., Jacobson M.R., O’Brien F., Noble W., Till S.J., Hamid Q.A., Nouri-Aria K.T. (1999). Long-Term Clinical Efficacy of Grass-Pollen Immunotherapy. N. Engl. J. Med..

[48] Eng P.A., Borer-Reinhold M., Heijnen I.A., Gnehm H.P. (2006). Twelve-year follow-up after discontinuation of preseasonal grasspollen immunotherapy in childhood. Allergy.

[49] Jacobsen L., Niggemann B., Dreborg S., Ferdousi H.A., Reese M.E., Reed C.E., Marcoux J.P. (2007). Specific immunotherapy has long-term preventive effect of seasonaland perennial asthma: 10-year follow-up on the PAT study. Allergy.

[50] Pfaar O., Lou H., Zhang Y., Klimek L., Zhang L. (2018). Recent developments and highlights in allergen immunotherapy. Allergy.

[51] Incorvaia C., Mauro M., Makri E., Leo G., Ridolo E. (2018). Two decades with omalizumab: What we still have to learn. Biol. Targets Ther..

[52] Warner J.O. (2010). Omalizumab for childhood asthma. Expert Rev. Resp. Med..

[53] National Institute for Health and Clinical Excellence (2019). Benralizumab for Treating for Treating Severe Eosinophilic Asthma Eosinophilic.

[54] Abrams E.M., Becker A.B., Szefler S.J. (2018). Current State and Future of Biologic Therapies in the Treatment of Asthma in Children. Pediatr. Allergy Immunol. Pulmonol..

[55] Gore C., Griffin R., Rothenberg T., Tallett A., Hopwood B., Sizmur S., O’Keeffe C., Warner J.O. (2016). New patient-reported experience measure for children with allergic disease: Development, validation and results from integrated care. Arch. Dis. Child..

[56] Warner J.O., Spitters S. (2017). Integrating Care for Children with Allergic Diseases: UK experience. Curr. Allergy Clin. Immunol..

